# Establishment and validation of nomogram model for the diagnosis of AFP-negative hepatocellular carcinoma

**DOI:** 10.3389/fonc.2023.1131892

**Published:** 2023-02-20

**Authors:** Long Liu, Qi Wang, Xiaohong Zhao, Yuxi Huang, Yuyi Feng, Yu Zhang, Zheping Fang, Shaowei Li

**Affiliations:** ^1^ Department of Hepatobiliary Surgery, Taizhou Hospital of Zhejiang Province, Zhejiang University, Linhai, Zhejiang, China; ^2^ Department of Hepatobiliary Surgery, Taizhou Hospital of Zhejiang Province, Wenzhou Medical University, Linhai, Zhejiang, China; ^3^ Department of Pharmacy, Taizhou Hospital of Zhejiang Province, Zhejiang University, Linhai, Zhejiang, China; ^4^ Department of Oncology, The First Hospital of the University of Science and Technology of China, Hefei, Anhui, China; ^5^ Department of Gastroenterology, Taizhou Hospital of Zhejiang Province, Linhai, Zhejiang, China

**Keywords:** alpha-fetoprotein, AFP-NHCC, nomogram model, early diagnosis, marker

## Abstract

**Introduction:**

As one of the most common malignant tumors in clinical practice, hepatocellular carcinoma (HCC) is a major threat to human health, where alpha-fetoprotein (AFP) is widely used for early screening and diagnoses. However, the level of AFP would not elevate in about 30-40% of HCC patients, which is clinically referred to as AFP-negative HCC, with small tumors at an early stage and atypical imaging features, making it difficult to distinguish benign from malignant by imaging alone.

**Methods:**

A total of 798 patients, with the majority being HBV-positive, were enrolled in the study and were randomized 2:1 to the training and validation groups. Univariate and multivariate binary logistic regression analyses were used to determine the ability of each parameter to predict HCC. A nomogram model was constructed based on the independent predictors.

**Results:**

A unordered multicategorical logistic regression analyses showed that the age, TBIL, ALT, ALB, PT, GGT and GPR help identify non-hepatic disease, hepatitis, cirrhosis, and hepatocellular carcinoma. A multivariate logistic regression analyses showed that the gender, age, TBIL, GAR, and GPR were independent predictors for the diagnosis of AFP-negative HCC. And an efficient and reliable nomogram model (AUC=0.837) was constructed based on independent predictors.

**Discussion:**

Serum parameters help reveal intrinsic differences between non-hepatic disease, hepatitis, cirrhosis, and HCC. The nomogram based on clinical and serum parameters could be used as a marker for the diagnosis of AFP-negative HCC, providing an objective basis for the early diagnosis and individualized treatment of hepatocellular carcinoma patients.

## Introduction

Liver cancer is one of the most common malignancies in clinical practice and is a major threat to human health. According to statistics, in 2023, liver cancer became the sixth-most common cancer worldwide and more than 900,000 new cases every year, besides, it is also the third leading cause of cancer-related death (1). In China, the occurrence of liver cancer accounts for half of the global value, with a high incidence and mortality rate (2). According to the pathological type, primary HCC can be divided into HCC, cholangiocellular carcinoma, and mixed HCC accounting for about 91.5% of cases. It is therefore necessary to accurately diagnose and treat HCC.

The levels of AFP, a 70-KD glycoprotein, decline rapidly after birth and remain low throughout the lifespan in normal physiology(3).In the 1960s, alpha-fetoprotein (AFP) was first used for the diagnosis and treatment of HCC (4). Roughly 30% to 40% of all patients with HCC will have negative serum levels of AFP, and 15% to 30% of patients with advanced HCC will initially have elevated serum AFP levels that subsequently return to normal values (5, 6). These factors make the diagnosis of HCC difficult, especially in cases of AFP-negative HCC, so there is an urgent need for more efficient diagnostic indicators to assist in the diagnosis of AFP-negative HCC in clinical practice. Generally, the early screening and diagnosis of AFP-negative HCC patients tend to rely heavily on imaging examinations, such as ultrasound, computed tomography (CT), and magnetic resonance imaging (MRI). Unfortunately, most AFP-negative HCC patients have small tumors at an early stage with atypical imaging features, making it difficult to distinguish benign and malignant nodules by relying on imaging examinations alone. further complicating matters is the fact that AFP-negative HCC lacks ideal biomarkers. Therefore, the misdiagnosis and underdiagnosis rates of AFP-negative HCC are both high (7).

And more importantly, diagnosis of AFP-negative HCC is meanful for clinical practice because the prognosis of these patients is poor than that of AFP-positive patients for poorly differentiated and rapid malignant progression (8–10),therefor, the clinical diagnosis of AFP-negative HCC has become an urgent issue hindering the early treatment and improved prognosis of HCC in general, so we urgently need to find new serum biomarkers other than AFP to facilitate early screening and the early diagnosis of AFP-negative HCC (11). At present, Serum biomarkers are non-invasive, convenient, economical, and reproducible diagnostic tools for oncology with accurate and repeatable measurements and a comparatively fast clinical turnaround time for detecting disease progression. In recent years, with the continuous development of technology, more and more serum biomarkers are used to diagnose AFP-negative HCC. For example, the microRNA miR-21 has higher serum values in patients with HCC compared with healthy subjects, and its positive rate in the AFP-negative HCC group was 77.6%, with a sensitivity of 81.2% and specificity of 83.2% (12). Vitamin K-deficient prothrombin II (PIVKA-II), which is significantly more strongly expressed in early HCC than in chronic hepatitis B, has a sensitivity of 76.3% and a specificity of 89.1% for the diagnosis of AFP-negative HCC, besides, PIVKA-II levels were associated with poor prognosis (13, 14). High-sensitivity AFP-L3%, with a sensitivity of 41.5% and a specificity of 85.1% for the diagnosis of AFP-negative HCC, and its combination with PIVKA-II can effectively improve the diagnostic value of AFP-negative HCC, and in the AFP-negative HCC group, the positive rate of these markers combined to detect early HCC was 81.8%, that to detect small HCC was 86.7%, and that to detect single tumors was 91.7% (15, 16). In addition, it has also been reported that circulating hematopoietic stem cells and cancer stem cells exploring new ideas for the diagnosis of liver cancer (17). Besides, independ on AFP, some other indicators shown outstanding diagnostic value, for example, STC2 was upregulated in both tumors and serum of HCC patients, and has good diagnostic significance and could be used as a co-biomarker for AFP to detect early HCC (18). the positive predictive value (PPV) of APEX1 was significantly higher than that of AFP (67.91% *vs*. 55.22%), and is a better biomarker for HCC diagnosis and prognosis than alpha-fetoprotein (19). However, while these new biomarkers are effective in diagnosing AFP-negative HCC, their expense and complexity make them difficult to employ in clinical practice on a large scale.

In contrast, routine serum tests supply a large source of data containing a great deal of disease-associated information that can provide diagnostic and prognostic decisions for diseases. For example, routine laboratory tests, such as evaluations of serum Prealbumin (PAB), a sensitive indicator of liver impairment and function (20), D-dime r(21), and γ-glutamine transpeptidase (GGT),a surface enzyme involved in glutathione metabolism, (22) and transaminases(ALT/AST) reflected damage of hepatocytes (23) are valuable for the diagnosis of AFP-negative HCC (20, 24). Besides, Due to the lack of diagnostic sensitivity and specificity of a single biomarker for AFP-negative HCC, combinations of multiple biomarkers are often used to effectively improve diagnostic efficiency. Huang et al. found that the fibrinogen/PA ratio and GGT/platelet ratio were much more powerful for the detection of AFP-negative HCC in combination than when applied alone (25). Several studies thus far have investigated the value of inflammatory response markers in the prognosis of HCC, but information on the diagnostic value in patients with AFP-negative HCC is lacking. These drove us to think about whether new meaningful findings could be obtained from the existing examination results. In the present study, we explored the diagnosis of AFP-negative HCC using conventional laboratory examination data in combination with several biomarkers, focusing on γ-glutamine aminotransferase-to-PA ratio (GPR) and γ-glutamine aminotransferase-to-glutathione aminotransferase (GAR), and clinicopathological indicators to assess their feasibility as predictive markers for patients with AFP-negative HCC.

## Materials and methods

### Collection of clinical specimens

This study collected and retrospectively analyzed 597 patients diagnosed with viral hepatitis B-associated liver disease in Taizhou Hospital, Zhejiang Province between January 2015 and December 2020, including 193 patients (32.3%) in the liver cancer group, 200 patients (33.5%) in the cirrhosis group, and 204 patients (34.2%) in the hepatitis group. In addition, 201 patients who visited the health checkup center during this period but did not suffer from hepatitis B viral hepatitis-associated liver disease were collected, and this group of patients was considered the healthy subject group.

1. Diagnostic criteria for HBV positive hepatitis: (1) positive HBsAg, or abnormal liver biochemistry (predominantly elevated serum alanine aminotransferase (ALT) and aspartate aminotransferase (AST)(WS 299-2008)

2. Diagnostic criteria for cirrhosis: (1) liver histopathology shows diffuse liver fibrosis and pseudolobular formation, (2) if liver histopathological examination is not performed, meet more than 2 of the following 5 and exclude non-cirrhotic portal hypertension can be clinically diagnosed as cirrhosis: (1) gastroscopy shows esophageal and gastric varices; (2) Imaging examination: ultrasound, CT or MRI have imaging features of cirrhosis; (3) Liver elasticity determination: LSM> 13kPa; (4) Decreased liver synthetic function: decreased serum albumin, prothrombin time prolonged; (5) Hypersplenism: platelets, white blood cells or hemoglobin decreased.(2019 Chinese Medical Association Hepatology Branch)

3. Diagnostic criteria for AFP-negative HCC: (1) all patients in the current study had serum AFP <10 ng/ml and were considered AFP-negative. (2) HCC patients were newly diagnosed with HCC, by pathological tests after hepatectomy or liver puncture tissue(26).

The exclusion criteria were as follows: (1) With other digestive system diseases; (2) Those with malignant tumors other than HCC; (3) with hematological or immune-related diseases; (4) recurrence of HCC after the first treatment (including surgical resection, radiofrequency ablation, etc.); (5) cases with incomplete data or missing data.

### Data acquisition

This was a retrospective clinical study, and all data were obtained from the electronic medical records of patients in Taizhou Hospital. Collected data included the gender, age, AFP, total bilirubin (TBIL), alanine transaminase (ALT), glutathione transaminase (AST), albumin (ALB), prothrombin time (PT), GGT, and PA values. The tumor size and number were determined by postoperative pathological tests. The liver function was assessed according to the Child-Pugh score standard. CNLC (China liver cancer staging) staging was performed for patients with HCC according to the “Diagnostic and treatment protocol for primary liver cancer (2019 edition) (27). The GPR value was calculated as GGT/PA, and the GAR value was calculated as GGT/AST, and most patients with hepatitis, cirrhosis and liver cancer were HBV-positive.

### Statistical analyses

The SPSS 27.0 (SPSS for Windows, version 22.0; SPSS, Inc. Chicago, IL, USA) and R4.1.0(MathSoft.USA) software programs were used for analyses and data processing. (1) Spearman’s correlation analysis was used to test the correlation between parameters, and redundant parameters with autocorrelation coefficients >0.7 were excluded. (2) Numerical variables that obeyed a normal distribution were statistically described as the mean ± standard deviation, and a t-test was used for comparisons between two groups, while an analysis of variance was used for comparisons among three or more groups. Non-normally distributed numerical variables were described as the median (interquartile spacing), and the comparisons between two groups was performed by Mann-Whitney U test, while comparisons among three or more groups was performed by Kruskal-Wallis H test. Categorical variables were analyzed using the chi-square test or Fisher exact test.(3) Unordered multicategorical logistic regression analyses were used to further analyze the efficacy of each parameter in identifying patients with non-hepatic and digestive disease, hepatitis, cirrhosis, and HCC. (4) Univariate and multivariate binary logistic regression analyses were used to determine the ability of each parameter to predict HCC. A nomogram model was constructed using by R4.1.0 (rms package) based on the independent predictors from the multivariate logistic regression analyses. (5) The Hosmer-Lemeshow test, calibration curve, decision curve, clinical impact curve, receiver operating characteristic (ROC) curve, and its area under curve (AUC) were used to verify the fit, stability and clinical value of the model. (6) Finally, the model was tested internally using 10-fold cross-validation and brought into the validation group for external testing.

## Results

### General clinical characteristics of 798 patients

According to the inclusion and exclusion criteria, a total of 798 patients were included in the study, including 193 patients (24.19%) in the liver cancer group, 200 (25.06%) in the cirrhosis group, 204 (25.56%) in the hepatitis group, and 201 (25.19%) in the healthy group. There was no significant difference in the general clinical information between the training group validation groups (*P*>0.05), indicating that the grouping was randomized and reasonable. More baseline information is shown in **Table 1**.

**Table 1:** General clinical characteristics of 798 patients.

Characteristics	Primary Cohort				Validation Cohort				*P value*
	HP (n=129)	CH (n=133)	LC (n=136)	HCC(n=134)	HP (n=64)	CH (n=66)	LC (n=69)	HCC(n=67)	
**Gender(male**/female**)**	60/74	96/40	86/47	96/33	29/38	48/21	43/23	48/16	0.938
**Age**	61(51, 66)	46(39, 54)	58(53, 65)	59(52, 65)	64(51, 72)	49(38, 57)	56(48, 65)	58(50, 66)	0.517
**TBIL**(μmol/L)	14.9(11.0, 18.8)	14.1(10.5, 17.7)	27.3(16.3, 60.8)	14.5(11.4, 18.8)	14.2(10.6, 18.0)	13.4(10.1, 19.5)	27.1(15.8, 49.6)	13.7(9.6, 17.1)	0.334
**ALT**(U/L)	20.0(13.8, 26.0)	65.0(27.0, 195, 3)	23.0(17.0, 33.0)	28.0(20.0, 45.0)	18.0(12.0, 25.0)	49.0(21.5, 199.5)	26.0(17.0, 36.0)	27.5(20.0, 43.5)	0.175
**AST**(U/L)	23.0(20.0, 29.0)	44.5(26.0, 96.0)	36.0(27.0, 56.0)	32.0(25.0, 48.0)	23.0(20.0, 27.0)	38.0(24.0, 90.0)	42.5(26.8, 55.3)	32.5(24.0, 43.0)	0.341
**ALB**(g/L)	44.9(41.6, 47.1)	42.3(38.9, 45.5)	30.4(25.7, 38.0)	39.8(37.0, 44.1)	44.4(41.2, 47.2)	41.5(39.6, 44.8)	31.7(25.9, 39.5)	39.9(36.9, 43.0)	0.664
**PT**(s)	13.2(12.3, 13.6)	14.3(13.6, 14.7)	16.6(14.6, 19.9)	14.0(13.3, 14.7)	13.2(11.8, 14.0)	14.3(13.5, 14.8)	16.7(14.4, 19.1)	14.0(13.2, 14.7)	0.827
**GGT**(U/L)	19.0(15.0, 27.3)	29.0(19.0, 47.8)	25.0(16.0, 41.0)	42.0(27.0, 72.0)	18.0(14.0, 23.0)	29.0(20.0, 50.0)	24.0(15.0, 47.5)	54.5(35.3, 97.8)	0.580
**PA**(mg/dl)	29.10 ± 6.34	20.31 ± 6.71	10.92 ± 8.03	18.25 ± 6.68	28.68 ± 7.40	19.75 ± 7.43	11.63 ± 7.38	18.88 ± 7.09	0.949
**GPR**	0.69(0.52, 0.92)	1.43(0.86, 2.59)	3.24(1.85, 5.95)	2.90(1.85, 5.07)	0.61(0.53, 0.81)	1.64(0.91, 2.93)	3.25(1.61, 6.00)	3.67(1.86, 7.20)	0.761
**GAR**	0.80(0.63, 1.10)	0.65(0.40, 0.91)	0.67(0.43, 1.01)	1.23(0.81, 1.89)	0.78(0.64, 1.00)	0.74(0.44, 1.21)	0.60(0.34, 1.18)	2.00(1.14, 2.63)	0.090

### Efficacy of serum parameters to discriminate patients with non-liver disease, hepatitis, cirrhosis, and liver cancer

Spearman’s correlation analysis showed that the autocorrelation coefficients of ALT and AST, ALB, and PA were all >0.7, so the redundant parameters AST and PA, which had poor discriminatory ability, were excluded. A univariate analysis showed that the gender, age, TBIL, ALT, PT, GGT, ALB, GPR, and GAR values had group differences, with ALB showing a significant difference among all four groups. The parameters with differences in the univariate analysis were included in the unordered multicategorical logistic regression analysis, and the age, TBIL, ALT, ALB, PT, GGT, and GPR all showed significant differences. Compared to patients with non-liver disease, PT, GGT, and GPR showed significant differences in the hepatitis, cirrhosis, and HCC groups. TBIL showed significant differences in the non-liver disease, hepatitis, and cirrhosis groups compared to patients with liver cancer. This suggests that the age, TBIL, ALT, ALB, PT, GGT, and GPR are useful for identifying patients with AFP-negative non-liver disease, hepatitis, cirrhosis, and HCC but are of limited value (Shown in **Table 2**).

**Table 2:** Efficacy of serum parameters to discriminate patients with non-liver disease, hepatitis, cirrhosis, and liver cancer.

Characteristics	Univariate χ^2^	Univariate *P*	Multivariate χ^2^	Multivariate *P*
**Sex(male**/female**)**	46.44	<0.001	6.61	0.086
Age(year)	132.48	<0.001	36.07	<0.001
**TBIL**(μmol/L)	152.04	<0.001	10.99	0.012
**ALT**(U/L)	166.12	<0.001	74.34	<0.001
**ALB**(g/L)	276.25	<0.001	37.42	<0.001
**PT**(s)	323.41	<0.001	76.44	<0.001
**GGT**(U/L)	178.28	<0.001	33.48	<0.001
**GPR**	378.47	<0.001	136.86	<0.001
**GAR**	153.80	<0.001	2.93	0.403

### Nomogram model based on serum parameters for predicting AFP-negative HCC

**Figure 1:**
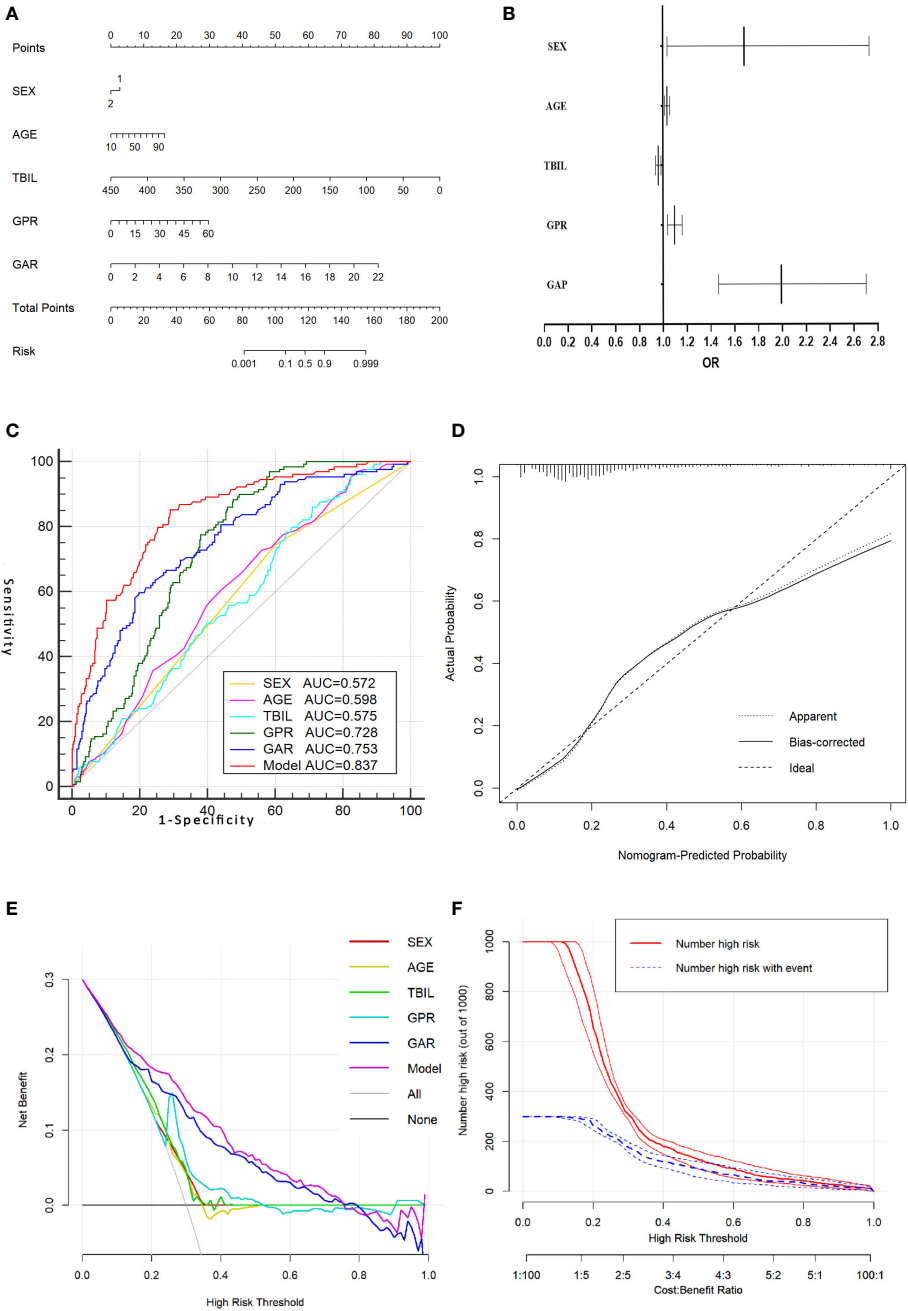
Nomogram model for predicting AFP-negative liver cancer and its evaluation curve. **(A)**.Nomogram model for predicting AFP-negative liver cancer. **(B)**. A forest plot of the OR values of each parameter of the Nomogram model. **(C)**. The ROC curve of the Nomogram model and the parameters in the Nomogram model. **(D)**. The calibration curve for the Nomogram model. **(E)**. The decision curve of the Nomogram model. **(F)**. The clinical impact curve of the nomogram model.

In the prediction of HCC, PA had greater diagnostic efficacy than ALB, so the redundant parameter excluded was ALB. In the training group, the univariate analysis revealed significant differences in the gender, age, TBIL, PA, GGT, GAR, and GPR between the HCC and non-HCC groups. Further one-way and multi-way logistic regression analyses were performed in which the gender, age, TBIL, GAR, and GPR were independent diagnostic factors for AFP-negative HCC patients (**Table 3**), resulting in the generation of a nomogram model with an AUC of 0.837. The *P* value of Hosmer-Lemeshow test was 0.120, indicating that the model was not overfitted. Internal validation was performed using 200 ten-fold cross-validation (mean AUC: 0.837) and 1000 resampling Bootstrap tests (mean AUC: 0.838), indicating a more stable model. The model was further brought into the validation group for external testing with an AUC of 0.840, indicating that the model was efficient and reliable. The calibration curve, decision curve, and ROC curve of the model are shown in **Figure 1**. The nomogram model we establish can efficiently and easily calculate the patient’s risk score. By calculating the sum of the scores of the parameters in the model, the patient’s cancer risk can be derived. In particular, **Figure 1A** intuitively shows the parameters and weights in the model, as well as the corresponding liver cancer risk, which provides new ideas and methods for the diagnosis of AFP-negative HCC.

**Table 3:** Univariate and multivariate analysis to identify independent influencing factors affecting the diagnosis of AFP-negative hepatocellular carcinoma.

Variables	Univariate analysis			Multivariate analysis		
	OR	95%CI	P	OR	95%CI	P
Gender	1.935	1.243-3.014	0.003	1.682	1.035-2.733	0.036
Age	1.031	1.013-1.049	0.001	1.034	1.014-1.055	0.001
TBIL	0.970	0.952-0.989	0.002	0.959	0.937-0.982	0.001
PA	0.979	0.959-1.000	0.050			
GGT	1.016	1.010-1.022	<0.001			
GPR	1.067	1.026-1.110	0.001	1.096	1.037-1.160	<0.001
GAR	2.561	1.909-3.437	<0.001	1.991	1.466-2.704	0.001

## Discussion

In recent years, the diagnosis of hepatitis, cirrhosis and HCC based on haematological parameters, such as γ-glutamyl transpeptidase/platelet ratio predicted liver fibrosis in patients with chronic hepatitis B (28). AST to platelet ratio index for the diagnosis of cirrhosis in patients with autoimmune liver disease (29). There was a significant correlation between serum AFP, GGT and TK1 levels and their clinicopathological and diagnostic value in patients with HCC (30), showing the feasibility of serum indicators in liver disease to play a diagnostic effect, but most of these studies were to study the correlation between healthy people and one of the pathological states in hepatitis, cirrhosis or HCC, clinical diagnosis is actually always a complex group which contained three disease states, our research focuses on the diagnosis of AFP-negative HCC in a mixed population of patients with three liver disease states, which may have a better clinical application.

GGT is widely recognized to play a role in the growth and development as well as the acquisition of resistance to drug toxicity in HCC(31, 32). GGT levels are reportedly elevated in HCC, regardless of AFP levels (33, 34), indicating that GGT levels are not affected by AFP and may thus be a new diagnostic and prognostic predictor of HCC, independent of AFP. This was precisely the reason we chose GGT as the focus of the present study.

However, these previous studies showed that GGT levels were significantly elevated in liver cancer compared to healthy individuals and only predicted the correlation between GGT and the liver cancer prognosis. The studies did not perform an in-depth study analysis of the diagnosis of AFP-negative HCC, so our current study examined the differences in the expression of GGT in diverse populations (healthy individuals, hepatitis, cirrhosis, and HCC). The samples included in our study are more complete than in previous studies, and the error caused by the bias in the population type has been minimized.

PAB was therefore selected as another predictive marker in the present study (35). However, several conditions are known to affect PAB levels, including cirrhosis, viral hepatitis, liver dysfunction, and an abnormal nutritional status. Therefore, even though PAB is useful as a regulatory indicator of hepatocytes, it is limited by its low diagnostic efficacy in detecting HCC.

Previous studies have also used PAB combined with the D-dimer and fibrinogen levels to diagnose AFP-negative HCC(7): PAB (AUC=0.900), PAB+D-dimer (AUC=0.941), and PAB+fibrinogen (AUC=0.901). However, the control group in those studies only included healthy individuals, not taking into account the fact that, in real clinical practice, physicians deal with a diverse group of patients, which can include those with hepatitis and cirrhosis. Therefore, in the present study, we selected a larger sample size, covering a wider range of patients, which better conformed to the actual situation in clinical practice. In the clinical setting, the most difficult part of diagnosing AFP-negative HCC is excluding patients with hepatitis and cirrhosis.

Serum GGT combined with the AST/ALT and GGT/ALT ratios have been shown to be of great value in predicting the prognosis of HCC (34, 36, 37). However, the utility of the GGT/AST for diagnosing AFP-negative HCC patients has not been reported. In our study, significant differences in the GGT/AST were found among healthy individuals and hepatitis, cirrhosis and HCC patients; furthermore, the results of univariate and multifactorial analyses showed that the GGT/AST could be an independent influential factor in the diagnosis of AFP-negative HCC.

In addition, in our present study, we constructed a nomogram model for the diagnosis of AFP-negative HCC by combining the independent influencing factors of gender, age, TBIL, GAR and GPR, which was further refined with univariate and multivariate analyses of each index. Our nomogram model showed a high performance in the diagnosis of AFP-negative HCC in both training (AUC=0.838) and validation (AUC=0.840) sets. Few previous studies have focused on the construction of a nomogram model based on serological indicators for the diagnosis of AFP-negative HCC, with most limited to the analysis of single- or several-factor ROC diagnostic curves showing incomplete consideration of factors and a single study sample of diseases that may bias the results(30, 37, 38).

In recent years, new advances have been made in the diagnosis of HCC and AFP-negative HCC by serological examinations, and new indices, such as, GP73, AFP-L3 and PIVKAII, have been used in clinical practice. According to relevant studies, the AUC values of GP73, AFP-L3 and PIVKAII for differentiating AFP-negative HCC from controls were 0.7811, 0.6094 and 0.856, respectively (13, 39), which were lower or only comparable to our present study model. Another retrospective study showed that the detection rate of the combined PIVKAII and AFP-L3 assay in patients with AFP-negative HCC was only 68.4%(39), which was lower than that of the combined GPR and GAR assay, and the sensitivity was only 40% when the results of the combined AFP-L3 and GP73 assay were used, which was inferior to the combined GPR and GAR. Considering the early occult, insidious nature of AFP-negative HCC and the economic burden of additional marker testing, combined with the present results, the present study model may aid in the preoperative noninvasive diagnosis of AFP-negative HCC and provide an early, effective, noninvasive and simple diagnostic marker for patients with occult HCC. In the clinical setting, it has the advantages of simple operation and economic ease of use.

Several limitations associated with the present study warrant mention. First, this was a single-center retrospective study, and the conclusions drawn need to be validated by multicenter randomized controlled and prospective research trials. Second, the relatively small sample size and the fact that all data originated from a single hospital may have biased the detection of these indicators. In addition, relevant information, such as the family history, was not obtained, and the distribution of different characteristics of patients with HCC increased the heterogeneity, which may have affected the final results. In a subsequent study, we will perform analyses of multiple medical centers on a larger scale, and more detailed information will be obtained to validate these results. In addition, in recent years, Numerous new nanoparticles have been applied to diagnosis and treatment of HCC and demonstrated unique advantages, for example, exosome miR-21 levels in the blood of HCC patients were significantly higher than in chronic hepatitis B (CHB) patients or healthy people and could be used as a potential diagnostic marker for HCC and might later become a non-invasive liquid biopsy marker for HCC(40). Synthetic nanoparticles have also shown outstanding performance in the diagnosis and treatment of HCC, for example, a new material system composed of glucose and TEMPO (2,2,6,6-tetramethylpiperidin-1-yl) oxide was designed to wrap the therapeutic drug CUDC101 and IR780, which play multiple effects such as treatment and multimodal imaging for HCC(41, 42). We consider whether our nomogram model can be combined with these nanoparticles to open up novel and more effective ideas for the diagnosis of AFP-negative HCC.

## Conclusions

Serum parameters can reveal, to some extent, intrinsic differences between non-hepatic disease, hepatitis, cirrhosis, and HCC(majority of patients were HBV-positive Chinese). The nomogram model based on clinical and serum parameters can be used as a marker for the diagnosis of AFP-negative HCC, providing an objective basis for the early diagnosis and individualized treatment of HCC patients.

## Data availability statement

The original contributions presented in the study are included in the article/supplementary material. Further inquiries can be directed to the corresponding authors.

## Ethics statement

The studies involving human participants were reviewed and approved by The Ethics Committee of Taizhou Hospital of Zhejiang Province, approved No.K20210519. The patients/participants provided their written informed consent to participate in this study.

## Author contributions

Conceived and designed the research: SL, YZ and ZF. Performed the clinical data collection: LL, QW, XZ. Analyzed the data: LL, YZ. Contributed data analysis: SL, ZF, YZ, YF. Wrote the manuscript: LL, QW. Responsible for the editing: SL and ZF. All authors contributed to the article and approved the submitted version.
